# Bilateral 4-quadrant laparoscopic-assisted transversus abdominis plane block reduces early postoperative pain after laparoscopic cholecystectomy: A prospective, single-blind, randomized study

**DOI:** 10.15537/smj.2023.44.2.20220407

**Published:** 2023-02

**Authors:** Eralp Çevikkalp, Mustafa Narmanlı, Halil Özgüç, Gökhan Ocakoğlu

**Affiliations:** *From the Department of Anesthesiology and Intensive Care (Çevikkalp); from the Department of General Surgery (Narmanlı, Özgüç), Bursa Private Medicabil Hospital; and from the Department of Biostatistics (Ocakoğlu), Faculty of Medicine, Uludağ University, Bursa, Turkey.*

**Keywords:** laparoscopic cholecystectomy, pain management, postoperative analgesia, port-site infiltration, transversus abdominis plane block, regional anesthesia

## Abstract

**Objectives:**

To investigate the efficacy of bilateral 4-quadrant laparoscopic-assisted transversus abdominis plane (BLTAP) block in laparoscopic cholecystectomy (LC).

**Methods:**

This study was carried out at Private Medicabil Hospital, Bursa, Turkey, between September 2021 and March 2022. Patients were randomly divided into 4 groups (n=40, each): i) the standard analgesia (SA) group received the block and port-site infiltration with normal saline (NS); ii) the local anesthetic group received the block with NS and port-site infiltration of bupivacaine; iii) the unilateral laparoscopic-assisted transversus abdominis plane (ULTAP) block group received 2-quadrant block with bupivacaine on the right and NS on the left and port-site NS infiltration; and iv) the BLTAP block group received bilateral bupivacaine and port-site NS infiltration. Postoperative 1-, 3-, 6-, 12-, and 24-hours visual analog scale (VAS) pain scores at rest and during cough, opioid requirement, presence of nausea and vomiting, and satisfaction scores were recorded.

**Results:**

The one-hour VAS score at rest was lower in the BLTAP block group than in the SA and ULTAP block groups. The change in VAS score was higher in the SA group than in the BLTAP block group. During cough, the one-hour VAS score was lower in the BLTAP block group than in the SA group. There were no differences among groups in other parameters.

**Conclusion:**

Bilateral 4-quadrant laparoscopic-assisted transversus abdominis plane block technique is more effective than SA, local anesthetic infiltration, and ULTAP block in preventing early postoperative pain after LC.

**Clinicaltrials.gov No.:** NCT04641403

**L**aparoscopic cholecystectomy (LC) is a commonly carried out surgical procedure associated with moderate pain, particularly in the early period after surgery. Pain caused by LC is usually managed with standard intravenous (IV) analgesia in the form of paracetamol, nonsteroidal anti-inflammatory drugs, and low-dose opioid administration, depending on the level of pain. With the increase in the number of outpatient surgical procedures and the introduction of accelerated recovery protocols, various approaches, such as local anesthetic infiltration, regional anesthesia techniques, and different block interventions, have been increasingly used and are included in postoperative pain management guidelines.^1,2^

The transversus abdominis plane (TAP) block is carried out by blocking the T6-L1 somatic nerves innervating the anterior abdominal wall from the parietal peritoneum to the cutaneous tissue in the neurofacial space between the internal oblique muscle and transversus abdominis muscle.^3^ It was first carried out blindly by Rafi.^4^ Various techniques have been developed up to date for the TAP block. The block is commonly carried out under the guidance of ultrasound (US).^4^ When carried out under US guidance, the rate of local anesthetic delivery to the proper plane is higher. However, it also has drawbacks, such as the requirement for equipment, practitioner, experience, and a longer learning curve.^5,6^ Several studies, reviews, and meta-analyses have also demonstrated the efficacy of TAP block for reducing pain and opioid consumption after different abdominal surgeries.^7-9^

Laparoscopic-assisted TAP (LTAP) block was carried out in 2011 before laparoscopic nephrectomy as a technique that combines the advantages of both techniques and can be easily carried out by a surgeon.^10^ As a relatively simple and practical method, LTAP has been thought to have major advantages for post-LC pain management in terms of time, resources, and cost. However, randomized controlled studies carried out on this issue in different centers have yielded different results.^11,12^ The different results may be attributed to the differences in the techniques used (subcostal, lateral block, 2-quadrants, 4-quadrants). There are debates regarding the administration areas and number of block techniques. For example, it has been reported that only blocking the right side of the abdomen will be sufficient for LC.^13^ Moreover, the study by Elamin et al,^11^ reported that TAP block should be applied to both subcostal and lateral areas as bilateral 4 quadrants (bilateral 4-quadrant laparoscopic-assisted TAP [BLTAP] block). The results of these different studies have created a controversy on the use of unilateral laparoscopic-assisted TAP (ULTAP) and BLTAP blocks in LC cases.

In routine practice, our center employs an approach of gradual administration of IV paracetamol, nonsteroidal anti-inflammatory drugs, and, when necessary, IV tramadol for the management of post-LC pain. However, TAP block or port-site local anesthetic infiltration can also be used depending on the surgeon’s preference. Studies on this subject do not provide a clear answer regarding the efficacy of TAP block application over conventional pain applications and the quadrants that should be applied with this technique.^11,13^ This prospective randomized study aimed to determine the efficacy of BLTAP block over other postoperative analgesia techniques (conventional IV analgesia, port-site local anesthetic infiltration, and unilateral 2-quadrant TAP block) in LC cases.

## Methods

The study was designed as a prospective, randomized, controlled, and participant-blind study and carried out at the General Surgery, Anesthesiology, and Reanimation Departments of Private Medicabil Hospital, Bursa, Turkey, between September 2021 and March 2022 in adherence to the Declaration of Helsinki and the principles of Good Clinical Practice. The approval for the study was obtained from the Clinical Research Ethics Committee of Bursa Uludag University, Faculty of Medicine, Bursa, Turkey (no.: 2011-KAEK-26/55).

Patients aged 18-70 years with American Society of Anesthesiologists (ASA) scores of 1 and 2 who underwent conventional 4-quadrant LC were included in the study. Those who refused to participate in the study, who underwent emergency surgery or open cholecystectomy, whose surgery was converted to open surgery, who had a body mass index of >40 kg/m^2^, who developed major intraoperative complications, who had a history of severe allergy, who had chronic analgesic use, and in whom the TAP technique was not properly carried out were excluded from this study.

Patients were randomized to the groups immediately before surgery using a computer-generated random number table and the surgeon was informed regarding the technique to be used for each patient. Patient’s data were recorded on a previously prepared form, and there was no record in this form to suggest the group of the patient. Although the surgeon and surgical team were not blinded, the anesthesia team and clinical nurses who carried out and recorded the postoperative assessments were blinded to the group of patients. In all groups, postoperative pain management was carried out by teams (clinical nurses and anesthesia team) who were blinded to the patient group by following standard orders, and the surgeon was not involved in this process. These forms were collected by the research coordinator nurse immediately before discharge and kept until the end of this study. The executive team was blinded of the results until the end of this study.

Before starting this study, educational materials containing theoretical information, shape, and video on the regional anatomy and block administration were provided to the entire team who would carry out the block to standardize the level of knowledge. This study was started after the pre-application of 10 patients by the surgical team. All surgeries were carried out by 2 staff (HO and MN). Three 20 ml injectors were kept ready on the operating table for all patients undergoing LC included in this study, and normal saline (NS) or local anesthetic was withdrawn into the injector depending on the study group. In the BLTAP block group, the local anesthetic was diluted with saline and prepared as 40 ml of 0.25% bupivacaine (Marcaine, AstraZeneca). For the port-site injection of local anesthetic and ULTAP block, 20 ml of 0.5% bupivacaine (Marcaine, AstraZeneca) was used; thus, the total dose of local anesthetic was similar between the 2 groups.

**Figure 1:**
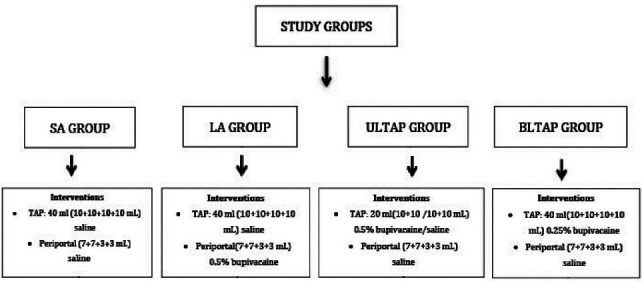
- After randomization, patients were assigned to each group and underwent the procedure. SA: standard analgesia, LA: local anesthesia, BLTAP: bilateral laparoscopic-assisted transversus abdominis plane block, ULTAP: unilateral laparoscopic-assisted transversus abdominis plane block

Patients were randomly assigned to the following groups: Group 1 (standard analgesia [SA] group, n=40): SA group received the block and port-site infiltration with NS. Group 2 (local anesthetic group, n=40): this group received the block with NS and port-site infiltration of anesthetic. Group 3 (ULTAP block group, n=40): following the port-site infiltration with NS, unilateral right-side 2-quadrant block (right subcostal and triangle of Petit) was carried out using bupivacaine, and left-side 2-quadrant block was carried out with NS. Group 4 (BLTAP block group, n=40): following port-site infiltration with NS, a bilateral 4-quadrant block (right and left subcostal and triangle of Petit) was carried out using bupivacaine. The administration techniques and dosages are shown in Figure 1.

**Figure 2:**
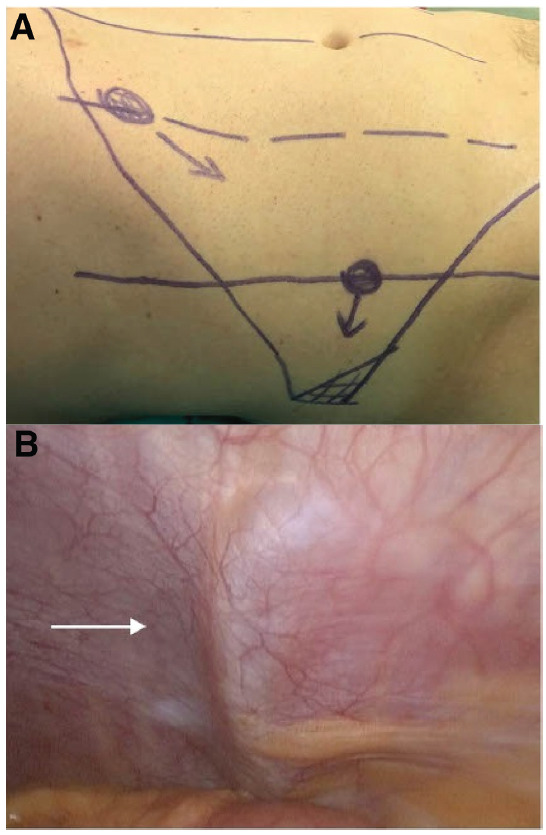
- Surface landmarks for: A) transversus abdominis plane block; and B) Doyle’s bulge after the injection of local anesthetic.

The first port was inserted through the umbilicus after the anesthesia was induced, and a standard pneumoperitoneum pressure of 12 mmHg was established. The block was carried out by first inserting a 22-G needle just above the iliac crest through the midclavicular line posteriorly into the triangle of Petit under the direct view of the laparoscopic camera. The needle was then inserted below the costal arch through the midclavicular line toward the anterior axillary line into the subcostal plane. The block needle was advanced under direct view until the 2 pops were felt, and the extraperitoneal space was accessed avoiding puncture of the parietal peritoneum. The needle was withdrawn about 0.5 cm to infiltrate the thin transversus abdominis muscle fibers in the area. The position of the needle tip in this area was confirmed by visualization of Doyle’s bulge and the spread of the local anesthetic.^11,14^ In the BLTAP group, TAP block was carried out at 4 points: bilateral subcostal area and bilateral triangle of Petit. In the ULTAP group, the blocks were applied at the same points (subcostal area and triangle of Petit) only on the right side (2-quadrant block). The procedure sites and administration techniques are shown in Figure 2.

Laparoscopic surgery was carried out using the conventional safety technique with 2 trocars sized 5-mm and 2 sized 5-mm. The pouch was removed through the epigastric port using an endobag. The 10-mm trocar sites were closed using absorbable sutures in the fascial plane.

After the transfer of patients to the operating room, an IV vascular access was established with a 22-gauge cannula, and 0.001 mg/kg midazolam was intravenously administered for sedation. Standard anesthesia monitoring included electrocardiogram (ECG), noninvasive blood pressure, peripheral oxygen saturation (SpO2), end-tidal carbon dioxide (ETCO2), and bispectral index ([BIS], Aspect 1000 Systems, Aspect Medical Systems Inc., Natick) measurements. For the induction of anesthesia, 0.2 mg/kg fentanyl (Talinat, Vem Ilaç, Ankara), 0.6 mg/kg rocuronium bromide (Esmeron, Merck Sharp Dohme, Istanbul), and 1-2 mg/kg propofol (Propofol, Polifarma, Tekirdağ) were induced by titration. The depth of anesthesia was maintained with a mixture of 50% O_2_ and 50% air to keep minimum alveolar concentration of sevoflurane as 2. To evaluate the depth of anesthesia, the BIS value was maintained between 40-60. Pressure-controlled mechanical ventilation was carried out by maintaining ETCO2 between 32-35 mmHg. Before the patients were awake, 1 g acetaminophen (Parol, Atabey Kimya, İstanbul) and 50 mg tramadol (Contramal, Grunenthal, Germany) were administered intravenously. Patients were then transferred to the general surgery ward when they could hold their heads straight for 5 seconds, with a 30-minutes modified Aldrete score above 8.

A standard protocol was followed for postoperative analgesia in all groups. Acetaminophen 1 g (Parol, Atabey Kimya, Istanbul) was gradually administered every 6 hours depending on the level of pain, while diclofenac sodium was intravenously administered every 12 hours when required. Intravenous tramadol 50 mg (Contramal, Grunenthal, Germany) was given as a rescue analgesic to patients with a pain score higher than 4.

Data on age, gender, height, weight, comorbidities, smoking status, length of hospital stay, operative time, intraoperative fluid volume, and complications of patients were collected using a standard form, after which intergroup comparisons were carried out. The primary outcome of this study was postoperative 1-, 3-, 6-, 12-, and 24-hours pain scores on the visual analog scale (VAS) during rest and cough.

The secondary outcomes included the requirement for rescue analgesics, pre-discharge patient satisfaction (1: strongly disagree, 5: strongly agree [based on a Likert-type scale]), presence of shoulder pain, nausea and vomiting, and discharge duration exceeding 24 hours.

### Statistical analysis

The mean postoperative one-hour pain scores on VAS and the rates of rescue analgesic use of 20 patients who underwent surgery prior to the study were determined. Based on the values obtained, it was determined that there should be 35 patients in each group, with a reduction in the pain scale score of 33% following LTAP block, at a significance level of 5% and a statistical power of 80%. In order to account for potential exclusions following randomization, this study included 40 patients per group. All patients gave informed consent. G*Power software was used to for power analysis.^15^

The consistency of normally-distributed continuous variables was examined with the Kolmogorov-Smirnov test. Continuous variables were presented as mean ± standard deviation (SD) or median (interquartile range [IQR]). If the variables followed a normal distribution, analysis of variance was carried out to make comparisons among groups; otherwise, the Kruskal-Wallis test was considered with the Dunn-Bonferroni test for pairwise comparisons. To compare VAS scores, the one-hour VAS scores were compared among groups; the difference in the VAS scores measured at 3-, 6-, 12-, and 24 hours compared to one-hour measurement was calculated; and these measurements were compared. In this way, the change in the scores (∆) obtained in the relevant periods according to the one-hour measurement was examined. Categorical variables are presented as numbers and precentages values and compared among groups using the Chi-squared or Fisher-Freeman-Halenda test. The Statistical Package for the Social Sciences, version 25.0 (IBM Corp., Armonk, NY, USA) was used for the statistical analyses.

## Results

**Figure 3:**
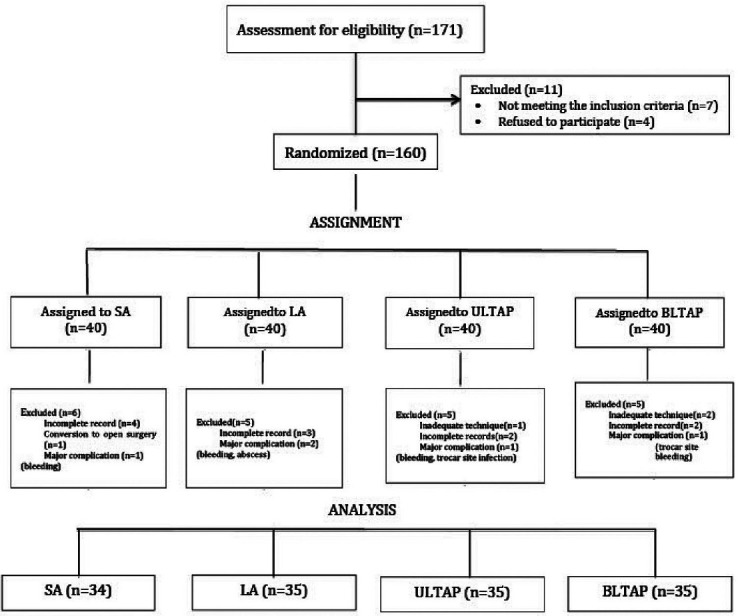
- The Consolidated Standards of Reporting Trials flow diagram. SA: standard analgesia, LA: local anesthesia, BLTAP: bilateral laparoscopic-assisted transversus abdominis plane, ULTAP: unilateral laparoscopic-assisted transversus abdominis plane

This study included 171 patients scheduled for LC. Eleven patients who did not meet the study criteria and refused to participate in this study were excluded from randomization. A total of 21 patients were excluded from the study for various reasons after randomization. Thus, 34 patients were included in the SA group and 35 patients in other groups. The Consolidated Standards of Reporting Trials diagram for this study is shown in Figure 3. The demographic, operative, and postoperative variables of the 3 groups are shown in Table 1. There were no statistically significant differences among groups in terms of these data (*p*>0.05).

**Table 1:** - Demographic intraoperative and postoperative secondary outcome results of the study.

Variables	SA group (n=34)	LA group (n=35)	ULTAP group (n=35)	BLTAP group (n=35)	*P*-values
Gender (male)	7 (20.5)	8 (22.8)	13 (37.1)	11 (31.4)	0.386^*^
Age (year)	46.5±12.9	50.0±17	49.4±11.8	51.2±15.0	0.574^†^
Height (cm)	166.4±9.1	167.3±11	168.4±8.9	166.3±9.4	0.784^†^
Weight (kg)	76.9±9.8	77.1±15.9	79.4±14.6	77.6±17.8	0.893^†^
Smoker	10 (29.4)	7 (20.0)	11 (31.4)	9 (25.7)	0.717^*^
***ASA class***					
1	19 (56.0)	19 (54.2)	19 (54.2)	18 (51.4)	0.942^*^
2	15 (44.0)	16 (45.7)	16 (45.7)	17 (48.5)	
Operative time (minute)	75.3±27.8	74.6±25.8	71.3±25.3	82.5±21.3	0.301^†^
Volume of fluid (ml)	583±293	545±252	605±192	560±261	0.767^†^
***Postoperative variables***					
Nausea and vomiting	9 (26.5)	6 (17.1)	10 (28.6)	9 (25.7)	0.697^*^
Rescue analgesic	10 (29.4)	6 (18.8)	9 (25.7)	3 (8.6)	0.149^*^
Shoulder pain	1 (2.9)	2 (5.7)	5 (14.3)	1 (2.9)	0.311^‡^
Satisfaction scale	4.42±1.03	4.69±0.47	4.77±0.42	4.81±0.39	0.051^†^
Discharge within 24 hours	26 (76.5)	25 (71.4)	29 (82.9)	26 (74.3)	0.713^*^

Values are presented as numbers and precentages (%) or mean ± standard deviation (SD). There were no significant differences among groups (p>0.05).

^*^ Chi-squared test,

^†^ analysis of variance,

^‡^ Fisher-Freeman-Halton test,

ASA: American Society of Anesthesiologists,

SA: standard analgesia,

LA: local anesthetic,

ULTAP: unilateral laparoscopic-assisted transversus abdominis plane,

BLTAP: bilateral 4-quadrant laparoscopic-assisted transversus abdominis plane

The analysis of the one-hour pain scale scores at rest revealed a statistical difference among the groups (*p*=0.002). The subgroup analyses showed that the BLTAP block group had a lower one-hour VAS score at rest compared to the SA block group (*p*=0.002) and the ULTAP block group (*p*=0.0245). The comparison of the changes in scores calculated by obtaining the difference according to the 1-hour score among the groups revealed a statistical difference in the change observed in the 24-hours score to the 1-hour score (*p*=0.033). The SA group had a higher change in VAS score compared to the BLTAP block group (*p*=0.038).

The 1-hour pain scores measured during cough were also significantly different among the groups (*p*=0.004). Based on the intergroup comparison, the 1-hour VAS score in the BLTAP group was lower than that in the SA group (*p*=0.002). The groups were similar in terms of the change in VAS scores from the 1-hour score. The VAS scores of all groups at rest and during cough are shown in Tables 2 & 3.

**Table 2:** - Postoperative visual analog scale scores at rest.

VAS scores	SA group (n=34)	LA group (n=35)	ULTAP block (n=35)	BLTAP block (n=35)	*P*-values^*^
one hour	2 (3)	2 (2)	2 (2)	1 (1)	0.002
	2.85±1.79	2.26±1.42	2.29±1.05	1.54±0.70	
3 hours	2 (2)	1 (1)	2 (1)	1 (0)	0.62
	2.09±1.29	1.66±1.03	1.66±0.77	1.14±0.73	
6 hours	1 (1)	1 (1)	1 (1)	1 (2)	0.066
	1.53±1.13	1.29±0.75	1.46±1.20	0.94±0.77	
12 hours	1 (1)	1 (1)	1 (2)	1 (1)	0.76
	1.15±0.99	0.91±0.74	1.17±1.18	0.83±0.62	
24 hours	1 (1)	1 (0)	1 (1)	1 (1)	0.47
	0.88±0.69	0.86±0.49	0.77±0.65	0.71±0.52	
∆_3hours→1hour_	-0.50 (1)	0 (1)	0 (1)	0 (1)	0.628
	-0.77±1.13	-0.60±1.09	-0.63±1.06	-0.40±0.85	
∆_6hours→1hour_	-1 (2)	-1 (2)	-1 (2)	-1 (1)	0.498
	-1.32±1.65	-0.79±1.65	-0.83±1.50	-0.60±0.98	
∆_12hours→1hour_	-1 (3)	-1 (2)	-1 (2)	-1 (1)	0.153
	-1.71±1.75	-1.34±1.70	-1.11±1.71	-0.71±0.86	
∆_24hours→1hour_	-1 (2)	-1 (2)	-2 (1)	-1 (1)	0.033
	-1.97±1.80	-1.40±1.56	-1.51±1.31	-0.83±0.86	
**Pairwise comparisons for VAS scores (one hour)**					
**P**_SA-LA_	**P**_SA-ULTAP_	**P**_SA-BLTAP_	**P**_LA-ULTAP_	**P**_LA-BLTAP_	**P**_ULTAP-BLTAP_
0.975	>0.999	0.002	>0.999	0.172	0.025
**Pairwise comparisons for VAS scores (∆**_**24hours→1hour**_ **)**					
**P**_SA–LA_	**P**_SA–ULTAP_	**P**_SA–BLTAP_	**P**_LA–ULTAP_	**P**_LA–BLTAP_	**P**_ULTAP–BLTAP_
0.969	>0.999	0.038	>0.999	>0.999	0.141

Values are presented as medians (interquartile ranges) and arithmetic mean±standard deviation (SD). The 1-hour VAS score was lower in the BLTAP group than in the SA (*p*=0.002) and ULTAP (*p*=0.0245) groups. In the comparison of the score changes calculated by taking the difference according to the 1-hour score among groups, the change was higher in the SA group than in the BLTAP group (*p*=0.038).

^∆^Difference score calculated according to the first-hour VAS score.

^*^ Kruskal-Wallis test.

VAS: visual analog scale,

SA: standard analgesia,

LA: local anesthesia,

BLTAP: bilateral laparoscopic-assisted transversus abdominis plane block,

ULTAP: unilateral laparoscopic-assisted transversus abdominis plane block

**Table 3:** - Postoperative visual analog scale scores during cough.

VAS scores	SA group (n=34)	LA group (n=35)	ULTAP group (n=35)	BLTAP group (n=35)	*P*-values^*^
one hour	3 (2)	3 (2)	3 (2)	2 (1)	0.004
	3.94±1.79	3.17±1.74	3.17±1.32	2.54±0.89	
3 hours	3 (2)	2 (1)	2 (1)	2 (0)	0.49
	2.91±1.36	2.69±1.60	2.37±1.09	1.94±0.84	
6 hours	2 (2)	3 (1)	2 (2)	2 (1)	0.68
	2.29±1.73	2.29±0.96	2.14±1.38	0.94±0.77	
12 hours	2(1)	2(1)	1(1)	1(1)	0.88
	1.88±1.47	1.83±0.99	1.83±1.29	1.43±0.74	
24 hours	1 (1)	1 (1)	1 (0)	1 (0)	0.83
	1.38±0.99	1.34±0.64	1.20±0.47	1.06±0.54	
∆_3hours→1hour_	-1 (2)	0 (1)	-1 (2)	-1 (1)	0.318
	-1.03±1.62	-1.03±1.60	-0.80±1.11	-0.60±0.74	
_∆6hours→1hour_	-1 (2)	-0 (1)	-1 (2)	-1 (1)	0.196
	-1.03±1.62	-0.49±1.60	-0.80±1.11	-0.60±0.74	
∆_12hours→1hour_	-2 (2)	-1 (2)	-2 (3)	-1 (1)	0.136
	-2.06±1.96	-1.34±2.14	-1.34±1.89	-1.11±1.32	
∆_24hours→1hour_	-2 (3)	-2 (3)	-2 (2)	-1 (1)	0.070
	-2.56±1.91	-1.83±1.89	-1.97±1.29	-1.49±0.92	
**Pairwise comparisons for VAS scores (one hour)**					
**P**_SA-LA_	**P**_SA-ULTAP_	**P**_SA-BLTAP_	**P**_LA-ULTAP_	**P**_LA-BLTAP_	**P**_ULTAP-BLTAP_
0.250	>0.999	0.002	>0.999	0.769	0.122

Values are presented as medians (interquartile ranges) and arithmetic mean±standard deviation (SD). The one-hour VAS score in the BLTAP group was lower than that in the SA group (*p*=0.002). In terms of the change in VAS scores from one-hour scores, there were no significant differences among groups.

^∆^Difference score calculated according to the first-hour VAS score.

^*^ Kruskal-Wallis test.

VAS: visual analog scale,

SA: standard analgesia,

LA: local anesthesia,

BLTAP: bilateral laparoscopic-assisted transversus abdominis plane block,

ULTAP: unilateral laparoscopic-assisted transversus abdominis plane block

In the routine practice of our center, IV 50 mg tramadol (Contramal, Grunenthal, Germany) was used as a rescue analgesic. Among all groups, only one dose was sufficient in the patient groups that required rescue analgesic use, and none of the patients required a second dose. There were no significant differences among the groups in terms of rescue analgesic use.

None of the groups developed postoperative vomiting, and there were no significant differences among groups in terms of the presence of nausea, patient satisfaction scores, complaints of shoulder pain, and discharge duration exceeding 24 hours. The secondary outcomes are shown in Table 1.

## Discussion

The results of this study comparing the efficacy of 4 different techniques in the management of post-LC pain demonstrated that the BLTAP block was more effective in preventing postoperative 1-hour pain compared to standard IV analgesia, port-site infiltration of local anesthetic, and the ULTAP block. However, there was no difference in the pain values measured subsequently and the use of rescue analgesics among the 4 techniques.

The efficacy of the LTAP block has been investigated using different surgical procedures. Field et al^14^ used this technique in their prospective randomized controlled study after laparoscopic ventral hernia repair and found that the technique reduced pain scores and opioid use. The efficacy of the LTAP block was first prospectively randomly tested by Elamin et al^11^ after LC. This study showed that the technique was more effective than port-site infiltration of local anesthetic in reducing pain scores in the first 6 hours, but this technique had no difference with port-site infiltration of local anesthetic in terms of opioid requirement.^11^ However, there are also different study results on the efficacy of the technique after LC. A recently published study by Siriwardana et al^12^ found an opposite result and showed that LTAP block did not decrease postoperative 6-hours pain scores and opioid use compared to local anesthetic administration. A prospective, randomized, and double-blind study by Houben et al^16^ investigating the efficacy of bilateral US-guided TAP block after LC, found that it had no advantage over SA in terms of parietal and visceral pain, but it decreased the requirement for intraoperative sevoflurane to some extent. Takimoto et al^17^ reported that the addition of a subcostal block to a lateral block increased the early analgesic efficacy in patients who underwent LC. Considering these studies, the local anesthetic injection was administered to 6 points in Fields et al’s study,^14^ whereas it was administered to 4 points in Elamin et al’s study.^11^ The block technique we used was the laparoscopic-assisted form of the US-guided 4-quadrant dual-block technique defined by Chen et al.^18^ These results suggest that the multi-point injection block is more effective in providing adequate analgesia.

Our study demonstrated that the BLTAP block was more effective in reducing postoperative 1-hour pain scores both during rest and cough compared to conventional IV analgesia and ULTAP block in LC cases. However, the BLTAP block had no difference with the other techniques in terms of opioid requirement for pain. Our study is similar to that of Elamin et al^11^ in terms of the techniques used and results produced. However, there are some differences with this study. First, our study comprised the SA, which is commonly used in clinical practice, and right-sided ULTAP block groups, which is another controversial topic. Another difference is that local anesthetic to the first port site and intraperitoneal area was not induced in all groups in our study. The main reason for this was to better observe the efficacy of the TAP block. Moreover, postoperative evaluation of visceral pain that is intended to be reduced by intraperitoneal administration is difficult and subjective. Simultaneously, the contribution of this type of pain to postoperative pain is minimal in laparoscopic procedures. This means that higher doses of local anesthetics should be induced.

Several studies have shown that local anesthetic agents have a more limited spread in isolated lateral and subcostal blocks; therefore, both sites should be blocked for complete and long-term blockade of the T6-L1 thoracolumbar nerves. The study of Carney et al^19^ investigating the spread of local anesthetic in the posterior, lateral, and subcostal approaches with magnetic resonance imaging showed that there was a more anterior spread in the lateral and subcostal injections, and that the blockade could be variable and nondermatomal. Similar results were also found in Støving et al’s study,^20^ suggesting that carrying out a lateral TAP block alone would not be an adequate approach, especially for midline incisions. The subcostal approach to be carried out more medially would provide a spread of local anesthetic agent in a more cephalad direction and could also block the segmental thoracic intercostal nerves. However, an isolated subcostal block alone may fail to block the lateral anterior axillary line.

The dual block technically combines the lateral/posterior TAP block with the subcostal approach, producing a block that involves both the upper and lower abdomen. It was first carried out by Borglum et al^21^ using the 4-point approach, and it was first called the 4-quadrant TAP block by Niraj et al.^22^ Borglum et al^21^ studied the dermatomal sensory anesthesia and spread characteristics of the US-guided 4-point injection technique that they used in 8 healthy volunteers. In this study, the lateral TAP block produced a dermatomal block from T10 to T12 and further extended the subcostal injection block up to T9-T7. Sondekoppam et al^23^ examined the lateral medial dual block in 9 cadaver series and found that the segmental spread was between T7-L1, similar to the results of Borglum’s study.^21^ It was concluded that the blockade of the lateral compartment alone was not significantly likely to anesthetize the lateral cutaneous nerve. Another study investigating the efficacy of port-site local anesthesia administration, SA, and US-guided unilateral TAP block after LC showed that unilateral TAP caused less pain and less tramadol use than the other 2 groups.^13^ However, no bilateral block group was included in this study. In addition, umbilical and epigastric port sites could often pass to the other side of the abdomen in LC procedures.^13^ Our study showed that the ULTAP block was ineffective in relieving pain. Therefore, we believe that bilateral administration would be more effective than unilateral administration.

Another controversial issue is the type of anesthetic agent induced. Liposomal bupivacaine has been indicated to be longer-acting and more effective. It is a relatively new agent, which is not always easily accessible. The addition of dexamethasone is another approach. Although several studies have shown that the efficacy of both procedures is extended, the results remain controversial.^24,25^ Laparoscopic cholecystectomy is a minimally invasive procedure, and the issue of pain is more significant in this procedure, especially in the first few hours. Our study demonstrated that the problem of pain in patients who received conventional analgesia was evident in the first hour, which was then significantly reduced. The SA group had a higher reduction in 24-hours pain score at rest versus the first hour compared to other groups. This result is attributed to the high initial pain score in the SA group and the significant reduction in pain score at 24 hours using all methods. The use of bupivacaine for the block provided sufficient effect in the first one hour. We believe that bupivacaine alone is sufficient for this surgical technique. The results of our study showed no difference in rescue analgesic use among the groups. None of the patients required a second dose. It is likely that LC is not an appropriate surgical method to compare the long-term effectiveness of block applications with the need for rescue analgesics. It would be more significant to investigate the effectiveness of block applications in these patients using other surgical methods.

### Study limitations

The surgeon carrying out the procedure could not be fully blinded in practice. However, this problem was attempted to be solved by standardizing postoperative pain management; blinding the team (anesthesia team and clinic nurses) implementing the pain management, assessments, and recordings of the patient group; providing these data to the data collectors after the discharge of the patient; and evaluating all data at the end of this study. Second, the study included only patients with ASA scores of 1 and 2, while those with ASA scores of 3 and 4 were excluded as they used several different drugs and the technique could pose difficulties in evaluating VAS scores. Nevertheless, this group of patients may be the subject of another study.

In conclusion, the results of this study suggest that bilateral 4-quadrant LTAP block is an easy-to-carry out and practical technique for reducing pain in the first hour after LC. Our study has the following most important result: the TAP block should be applied to the 4 quadrants to have a sufficient effect in preventing post-LC pain. The efficacy of the block on pain in later hours and rescue analgesic use should be investigated using different surgical methods. New studies comparing different block methods will provide more detailed information in determining the block method that is more effective and applicable.
